# Bio-efficacy, physical integrity, community usage and washing practices of mosquito nets treated with ICON MAXX long-lasting insecticidal treatment in India

**DOI:** 10.1590/0074-02760160287

**Published:** 2017-01-19

**Authors:** Sudhansu Sekhar Sahu, Kasinathan Gunasekaran, Kilakootil Narayanan Vijayakumar, Purushothaman Jambulingam

**Affiliations:** Vector Control Research Centre (Indian Council of Medical Research), Medical Complex, Indira Nagar, Pondicherry, India

**Keywords:** ICON MAXX, lambda-cyhalothrin, long-lasting insecticidal treatment of nets, LLIN treatment kit, Odisha, India

## Abstract

**BACKGROUND:**

New brands of potential long lasting insecticide nets (LLINs) and LLIN treatment kits require field evaluation before they are used in a vector control programme.

**OBJECTIVES:**

The aim of this study was to evaluate the bio-efficacy, usage, washing practice and physical integrity of nets treated with LLIN treatment kit, ICON MAXX in a phase III field trial in Odisha state, India.

**METHODS:**

A total of 300 polyester nets treated with ICON MAXX and 140 polyester nets treated conventionally with lambda-cyhalothrin CS 2.5% ITNs were distributed. The bio-efficacy was evaluated with WHO cone bioassay. The chemical analysis of netting pieces was done at the beginning, after 12 and 36 months of the trial.

**FINDINGS:**

After one year of distribution of nets, the bioassay showed 100% mortality on both ITNs and ICON MAXX treated nets. At 36 months, the overall pass rate was 58.8% and the mean lambda-cyhalothrin content of LLINs was 34.5 mg ai/m^2^, showing a loss of 44.4% of the original concentration.

**CONCLUSION:**

ICON MAXX treated LLIN was found to retain bio-efficacy causing 97% knockdown of *Anopheles stephensi* up to 30 months and met the WHOPES criteria. However, the desired bio-efficacy was not sustained up to 36 months.

Successful implementation of insecticide treated nets (ITNs) at community level for malaria control is hampered by several technical, operational, economic and social factors (Jambulingam et al. 2008). The rapid loss of bio-efficacy of ITNs due to washing, and low re-treatment rates, particularly in difficult-to-reach areas, limit the operational effectiveness of ITNs programme (Lines 1996, Jambulingam et al. 2008). To overcome these operational constraints, factory made long-lasting insecticidal nets (LLINs) and long-lasting net treatment kits have been advocated (WHO 2007a, Malima et al. 2008).

In the factory made LLINs, insecticide is either incorporated into the fibers at the time of yarn extrusion or coated around the fibers of the net. A long-lasting treatment involves dipping of a conventional net in aqueous suspension of an insecticide and a binder (WHO 2007a). Currently, the only long-lasting net treatment kit available is ICON MAXX.

In ICON MAXX, the insecticide is combined with a polymer resin that coats the netting fiber. The kit is based on the slow-release capsule suspension (CS) of lambda-cyhalothrin that was previously evaluated by World Health Organization Pesticide Evaluation Scheme (WHOPES) and was recommended for treatment of mosquito nets by the 11th meeting of the WHOPES Working Group (WHO 2007a). These kits include a dose of binder (usually a resin), which is mixed with the recommended volume of water prior to the insecticide is added. In the process of net drying after dipping in the insecticide solution, the resin polymerizes around the fibres, binding the insecticide. The use of such kits is a new strategy to provide a long-lasting treatment (WHO 2007b).

New brands of potential LLINs and kits providing long-lasting treatment require field evaluation before they are recommended for use in a vector control programme. In the current Phase III (large-scale) field trial, polyester nets treated with the new long-lasting treatment kit, ICON MAXX (hereafter reffered as LLINs) were evaluated against *Anopheles fluviatilis*, the major malaria vector in the study area in comparison with polyester nets conventionally treated with the same insecticide (lambda-cyhalothrin) (hereafter referred as ITN) in a *Plasmodium falciparum* endemic area of Odisha state in India in collaboration with the WHOPES to assess the bio-efficacy, usage, washing practice and physical integrity of nets over a period of three years.

## MATERIALS AND METHODS

*Study area* - The study was carried out during June 2011 to July 2014 in five villages namely Ledriguda, Ambliambaguda, Kindriguda, Niraniguda and Bageipadar of Laxmipur Community Health Centre (CHC) of Koraput district of Odisha state, which is predominantly inhabited by tribes.

*Study design* - Two types of nets were compared, i.e., polyester nets treated with ICON MAXX (lambda-cyhalothrin CS 10%) long-lasting net treatment at the dosage of 50 mg ai/m^2^ (research arm 1) and polyester nets of same colour and size treated conventionally with lambda-cyhalothrin (CS 2.5%) (research arm 2) at the WHO recommended dosage of 15 mg ai/m^2^ under field conditions. The ITN was evaluated for a period of one year while the LLIN was evaluated for three years. A census was carried out in the five study villages prior to the start of the study. A random list of ID numbers according to the sample size was prepared in SPSS software and allocated to LLINs or ITNs. A total of 440 households were selected for net distribution and were included in the study. Of these, 300 households were given one coded LLIN each, and another 140 households were given one coded ITN each in the beginning. To cover the remaining persons in the households, 395 non-coded ICON MAXX nets with an identification mark were distributed. All households who were included in the study and distributed with nets were not aware of which type of nets they had received. After one year of the study, all remaining ITNs were withdrawn and replaced with non-coded ICON MAXX nets. Full community coverage with an LLIN/ITN was ensured in order to obviate the need of other vector control interventions in the villages.

Before distribution, polyester nets of size 120 x 200 x 180 cm were treated with ICON MAXX according to the manufacturer’s instructions, using 7.3 mL of lambda-cyhalothrin and 7.7 mL of binder mixed in an appropriate amount of water. Other polyester nets were treated with lambda-cyhalothrin 2.5% CS to a target dose of 15 mg ai/m^2^. All nets were treated by dipping them in aluminium basins for two minutes, allowed to drip over the basin to remove excess water and then dried horizontally in the shade.

*Ethical approval and informed consent* - The study was approved by the Human and Animal Ethics committee of the Vector Control Research Centre, Pondicherry, India. Villagers were informed about the aims and objectives of the study through group meetings. A written informed consent was obtained from all heads of households enrolled in the study at the time of census during June 2011.

*Chemical analysis* - To determine insecticide content, netting pieces were cut at the beginning of the trial (for baseline), and after one year of the trial. From each research arm, 30 nets were destructively and randomly sampled for chemical content analysis. This was done by selecting 30 ID numbers each from LLIN and ITN arms. After 36 months of household use of LLINs, to confirm the availability of the insecticide in the used LLINs, 80 nets were destructively and randomly sampled for chemical analysis. For the chemical analysis, sampling was performed according to the scheme specified by WHOPES (WHO 2005, 2013). Thus, from each of the 30 or 80 sampled nets, four rectangular pieces of 30 cm x 30 cm size were cut from positions 2, 3, 4 and 5 using sharp scissors with position 1 excluded, as illustrated in the guidelines. The sub-samples were rolled up and placed in new, clean and labelled aluminium foil and dispatched to the Phytopharmacy Department of the Agricultural Research Centre in Gembloux, Belgium (a WHO Collaborating Centre) for chemical analysis.

*Insecticide efficacy evaluation* - Insecticidal effect of ITNs/ LLINs was evaluated using cone bioassays following WHO procedure (WHO 2005, 2013) at the beginning and at the end of every six months up to three years in the case of LLINs and up to one year in the case of ITNs. During each bioassay up to 30 months, IDs of 30 randomly selected nets of each type (LLINs/ ITNs) were withdrawn from the households and used for cone-bioassays. At 36 months, bioassays were carried out with 80 randomly selected coded LLINs. The coded ITNs or LLINs that were withdrawn each time for bioassays/chemical analysis were replaced with non-coded ICON MAXX nets without any identification mark. WHO cone bioassays were initially conducted using wild-caught *An. fluviatilis*, major malaria vector in the study area and were susceptible to synthetic pyrethroids (Sahu et al. 2014). Since, adequate number of mosquitoes of this species could not be collected due to low mosquito densities, as a result of mass distribution of LLINs in the study district during early 2013, beginning at 24 months, a susceptible strain of *An. stephensi* was used for bioassays.

A total of 1500 field collected semi-gravid mosquitoes (30 nets x 5 positions per net x 5 mosquitoes per piece x 2 replicates) were exposed to LLINs and another 1500 to ITNs during the bioassay conducted at the beginning of net distribution. Since, the density of *An. fluviatilis* was low in the field, only 750 adult females of this species could be exposed each time in the bioassay conducted at the end of six, 12 and 18 months of the study*.* At 24 and 30 months, a total of 1500 laboratory reared non-blood-fed, two-five-day-old *An. stephensi* females were exposed each time in the bioassay. At 36-month follow up, a total of 3200 laboratory reared non-blood-fed, two-five-day-old *An. stephensi* adults were exposed to 80 randomly selected LLINs, but only to net pieces from positions 2 to 5 according to the WHOPES sampling scheme (WHO 2005, 2013). Knockdown (KD) was recorded after 60 min and mortality after 24 h. Mosquitoes exposed to untreated nets were used as controls. The bioassays were done at 27 ± 2ºC and 75 ± 10% RH.

LLINs which caused a KD rate of < 95% and a bioassay mortality of < 80% were subjected to tunnel test in the laboratory as per WHO guidelines (WHO 2005, 2013). The tunnel test was used to measure the mortality and blood feeding inhibition of host-seeking mosquitoes in an experimental chamber. The assay was carried out in a laboratory by releasing non-blood-fed female anopheline mosquitoes aged five-six days into a 60-cm tunnel (25 cm x 25 cm square section) made of glass. At each end of the tunnel, a 25-cm square cage covered with polyester netting was fitted. The LN netting sample, held in a disposable cardboard frame, was placed at one third the length of the glass tunnel. A total of 100 female *An. stephensi* were introduced in a tunnel at 18:00 h. The following morning at 09:00 h, the mosquitoes were removed using a mouth aspirator and counted separately from each section of the tunnel and mortality and blood feeding rates were recorded. During the tests, the tunnels were maintained at 27 ± 2ºC and 75 ± 10% RH under subdued light. Two tunnels were used simultaneously, one with treated netting sample and other with untreated netting sample. Blood feeding inhibition was determined by comparing the proportion of blood-fed females (alive or dead) in treated and control tunnels. Overall, mortality was measured by pooling the mortalities of mosquitoes from the two sections of the tunnel.

If mortality in the tunnel test fell below 80% and blood feeding inhibition fell below 90%, the net was considered to have failed to meet WHOPES criteria (WHO 2005, 2013). The results of the cone and tunnel tests were considered together in judging net performance. A candidate net is deemed to meet the requirements for an LLIN if, at the end of three years, at least 80% of the sampled nets retain bio-efficacy in the WHO cone bioassay or the tunnel test as described in WHO guidelines (WHO 2005, 2013).

*Assessment of usage pattern, net washing practice and physical integrity of nets* - A team of experienced field staff interviewed the households using a structured pre-tested questionnaire to assess usage pattern, washing practice and physical integrity of nets. At the end of week one and months six, 18 and 30 after net distribution, heads of 30 randomly selected households were interviewed each time visiting door-to-door. Houses once surveyed for interview were excluded from the subsequent interviews. After 12 and 24 months of net distribution, survey was done covering the households with the remaining coded LLINs and at the end of 36 months, all the 300 households (120 remaining households with coded LLINs + 180 households with non-coded LLINs with identification mark distributed at the beginning of the study) were surveyed. Net usage pattern for year-round and every night was studied only from 12 months to 36 months at the interval of six months. All ITNs were replaced with LLINs after one year, the data pertaining to the usage pattern, net washing practice and physical integrity of nets was analysed for LLINs and discussed in this MS.

The nets given to the interviewed households were examined for presence of holes on them and if present, size (by measuring diameter in cm) and location of the holes, tears, burns and open seams were recorded. Based on the size, the holes were grouped into four categories, viz. Size 1: smaller than a thumb (0.5-2 cm); size 2: larger than a thumb but smaller than a fist (2-10 cm); size 3: larger than a fist but smaller than a head (10-25 cm); size 4: larger than a head (> 25 cm) and respectively weighted as 1, 23, 196 and 576 (WHO 2011, 2013). The hole index was calculated as: (1 x no. of size-1 holes) + (23 x no. of size-2 holes) + (196 x no. of size-3 holes) + (576 x no. size-4 holes) (WHO 2011, 2013). The hole area of single hole of size 1, size 2, size 3 and size 4 was calculated as 1.23, 28.28, 240.56 and 706.95, respectively (WHO 2011, 2013). The hole area of each net was calculated as: (1.23 x no. of size-1 holes) + (28.28 x no. of size-2 holes) + (240.56 x no. of size-3 holes) + (706.95 x no. size-4 holes) (WHO 2011, 2013).

*Statistical analysis* - All data were transferred from the data forms to excel sheet in the computer. Data were analysed using EPI DAT 3.1 SOFTWARE. For percentage (rates), exact Binomial distribution with 95% confidence intervals (95% CI) were used. For continuous variables the arithmetic mean was used depending on the distribution of values compared to a normal distribution. Mortalities of mosquitoes in cone bioassays were compared between ITNs and LLINs using Chi-square test at the beginning and after six months of the trial. Since, 100% mortality was observed with both ITNs and LLINs at 12 months, Chi-square test could not be performed. A p-value < 0.05 was considered as significant.

## RESULTS

The population of the five selected study villages was 1760 in 456 households. The villages were mostly (> 85%) inhabited by tribes (Paraja and Kondha). Their literacy rate was 38.5%. The houses were brick built with asbestos or tiled roof. Most of the villagers were labourers involved in agriculture. The houses were small with two rooms including one kitchen.

*Insecticide content* - Results of the chemical analysis done on base line samples showed that the average (95% CI) lambda-cyhalothrin content in ICON MAXX nets was 62.0 ± 7.4 mg/m^2^ (59.2-64.8). The target dose for the net provided in the trial (120 x 200 x 180 cm) was 50 mg/m^2^. The average (95% CI) lambda-cyhalothrin content in samples of ITNs was 20.6 ± 2.8 mg/m^2^ (19.6-21.6), which was also marginally higher than the target concentration of 15 mg/m^2^.

After one year of household use, relatively a lower concentration of lambda-cyhalothrin than the target concentration was found in both ICON MAXX LLINs and ITNs. The average (95% CI) lambda-cyhalothrin content in the LLIN was 45.2 ± 15.8 mg/m^2^ (39.3-51.1) with a range of 2.2 to 70.8 mg/m^2^ and in the samples of ITNs, the average insecticide content was 13.7 ± 11.8 mg/m^2^ (9.3-18.1) with a range of 0.4 to 64.7 mg/m^2^. The analysis further showed that after one year of household use, the mean lambda-cyhalothrin content decreased to 45.2 mg/m^2^ in LLIN corresponding to 72.9% of the baseline content and to 13.7 mg/m^2^ in the ITN corresponding to 64% of the lambda-cyhalothrin content at baseline.

The chemical analysis done with 50 LLINs after 36 months of field use showed that, the mean (95% CI) lambda-cyhalothrin content further reduced to 34.5 ± 20.4 mg/m^2^ (28.7-40.3) with a range of 3.0 to 78.8 mg/m^2^, which was lower than the target dose of 50 mg/m^2^ and this was corresponding to 55.6% of the baseline content.

*Bioassay* - In cone-bioassays conducted at the beginning of the trial, the mean mortality (95% CI) of *An. fluviatilis* was 92.3% (91.7-92.8%) and 88.8% (87.9-89.7%) against ITNs and LLINs, respectively (Table I). There was no significant difference (χ^2^ = 0.52; p = 0.47) between ITNs and LLINs indicating their similar effect. However, both ITNs and LLINs (60 nets) produced a KD of 100%. After six months of net distribution, the mortality was 99.7% (99.2-100.3%) and 100% respectively against ITNs and LLINs (Table I); the KD rate was 100% against both ITNs and LLINs. There was no significant difference (χ^2^ = 0.00; p = 0.97) in the mortality rate between ITNs and LLINs. After 12 months of net distribution, both ITNs and LLINs caused 100% KD and 100% mortality of *An. fluviatilis* indicating that the two types of nets had comparable residual efficacy at the end of one year (Table I). At 18 months, the LLINs showed 100% KD and 100% mortality of *An. fluviatilis* (Table I)*.* Cone-bioassays done at 24 months with susceptible *An. stephensi* showed a KD of 100% and a mean corrected mortality of 88.3% (85.8-90.7%) (Table I). All LLINs tested in bioassays up to 24 months passed the WHOPES criteria.

**TABLE I:** Summary of bioassays and tunnel tests conducted on ICON MAXX long-lasting net treatment and lambda-cyhalothrin ITNs

Survey (month)	ICON MAXX long-lasting net treatment						Lambda-cyhalothrin ITN					
	
	Cone bioassay			Tunnel tests			Cone bioassay			Tunnel tests		
			
	No. of nets tested	Mean knock-down (95% CI)	Mean mortality (95% CI)	No. of nets tested	Mean mortality (95% CI)	Mean blood-feeding inhibition (95% CI)	No. of nets tested	Mean knock-down (95% CI)	Mean mortality (95% CI)	No. of nets tested	Mean mortality (95% CI)	Mean blood-feeding inhibition (95% CI)
1	30	100.0	88.8 (87.9-89.7)	---	---	---	30	100.0	92.3 (91.7-92.8)	---	---	-
6	30	100.0	100.0	---	---	---	30	100.0	99.7 (99.2-100.3)	---	---	-
12	30	100.0	100.0	---	---	---	30	100.0	100.0	---	---	-
18	30	100.0	100.0	---	---	---	0	-	-	-	-	-
24	30	100.0	88.3 (85.8-90.7)	---	---	---	0		-	-	-	-
30	30	97.3 (95.3-99.4)	67.5 (57.7-76.3)	6	34.5 (13.8-55.2)	30.7 (9.9-51.4)	0	-	-	-	-	-
36	80	90.1 (88.5-91.7)	76.2 (73.1-79.2)	35	41.5 (34.9-48.1)	29.0 (24.1-33.9)	0	-	-	-	-	-

95% CI: 95% confidence intervals.

After 30 months of net distribution, the KD (95% CI) was 97.3% (95.3-99.4%) and the mortality was 67.5% (57.7-76.3%) (Table I). Out of the 30 LLINs tested, 24 (80%) passed the WHOPES criteria as these nets caused ≥ 95% KD or ≥ 80% mortality. The remaining six (20%) nets caused < 95% KD or < 80% mortality and as per the WHOPES guidelines; tunnel test was performed on these six nets (Table I).

After 36 months of household use, the overall KD (95% CI) was 90.1% (88.5-91.7%) and the mean mortality was 76.2% (73.1-79.2%) in the cone-bioassays (Table I). Out of 80 LLINs tested in bioassays, 45 (56.3%) passed the WHOPES criteria with a KD of ≥ 95% or a mortality of ≥ 80%. The remaining 35 (43.7%) nets caused < 95% KD or < 80% mortality and these 35 nets were subjected to tunnel test (Table I).

*Tunnel test* - In the tunnel tests carried out at 30 months with the six LLINs, the mean mortality (95% CI) was 34.5% (13.8-55.2%) and blood feeding inhibition was 30.7% (9.9-51.4%) (Table I). Thus, none of these LLINs passed the WHOPES criteria.

The tunnel testing of the 35 nets after 36 months of household use showed a mean mortality (95% CI) of 41.5% (34.9-48.1%) and a blood feeding inhibition of 29.0% (24.1-33.9%) (Table I). Among the 35 LLINs, two (5.7%) passed the WHOPES criteria (mortality ≥ 80.0% or blood feeding inhibition ≥ 90%) and the remaining 33 (94.3%) LLINs did not pass (Table II). The mortality of two nets passed in tunnel test was 80% and 90.9%. After combining the results of cone-bioassays and tunnel tests carried out after 36 months of net distribution, out of the 80 LLINs tested, 47 (58.8%) passed the WHOPES criteria (Table II).

**TABLE II:** % (No nets passing/no. nets tested) ICON MAXX and ITN meeting WHO efficacy criteria in cone and tunnel tests, and their combined pass rate

Survey (month)	ICON MAXX long-lasting treatment			Lambda-cyhalothrin ITN		
	
	Cone bioassays	Tunnel tests	Cone and tunnel tests combined	Cone bioassays	Tunnel tests	Cone and tunnel tests combined
1	100 (30/30)	-	100 (30/30)	100 (30/30)	-	100 (30/30)
6	100 (30/30)	-	100 (30/30)	100 (30/30)	-	100 (30/30)
12	100 (30/30)	-	100 (30/30)	100 (30/30)	-	100 (30/30)
18	100 (30/30)	-	100 (30/30)	-	-	-
24	100 (30/30)	-	100 (30/30)	-	-	-
30	80.0 (24/30)	0 (0/6)	80.0 (24/30)	-	-	-
36	56.3 (45/80)	5.7 (2/35)	58.8 (47/80)	-	-	-

*Usage of nets* - During the surveys conducted from 12 to 36 months, 66.7% to 93.3% of the households reported sleeping every night year-round (Table III). Zero to 7.3% households reported not using the nets citing one or other reasons. The reliability of the statements given by the respondents on net usage was verified by inspecting the position of the nets in the houses during the survey. At 36 months, majority of the nets (81.7%, n = 278) were found hanging either above the beddings or mattress. This could be an indirect evidence for the net usage by the people. However, a sizable percentage (18.4%, n = 278) of the people were keeping the nets inside boxes indicating seasonal or occasional use.

**TABLE III:** Usage of LLINs by the households

Net use	Percentage of use of LLINs after months of distribution				

	12 (n = 240)	18 (n = 30)	24 (n = 180 )	30 (n = 30)	36 (n = 300)
Year - round and every night	75.8	93.3	81.1	80.0	66.7
Year - round occasionally	7.9	0	8.9	6.7	20.7
Seasonally but every night	7.9	0	2.8	13.3	5.3
Seasonally and occasionally	4.6	6.7	1.7	0	0
Not using the net	3.8	0	5.6	0	7.3

*Washing practice* - At 12 months post-distribution, the survey showed that 79.6% nets were washed and the mean number of washes per LLIN was 1.8 ± 1.6 (range: 0-12) (Table IV). All nets were washed using cold water and locally available detergent powder; 52.4% LLINs were dried in sunlight, 34% in shade and 13.6% inside houses (Table V). After 24 months of distribution, 92.2% of nets were reported to be washed and the mean number of washes per LLIN was 4.8 ± 4.1 (range: 0-25) (Table IV). Nearly 90% nets were washed in cold water and the remaining in warm water; 4.8% nets were washed with soap, 94.6% with commercially available detergent powder and the remaining with locally available detergent soap. Maximum LLINs (72.9%) were dried in sunlight, 19.3% in shade and 7.8% inside houses (Table V). At 36 months, 89.3% nets were washed and the mean number of washes per LLIN was 8.3 ± 6.9 (range: 0-36) (Table IV). Commercially available detergent powder was used to wash 98.9% LLINs and detergent soap for the remaining. All nets were washed in cold water; 63.8% were dried in sunlight, 31.3% in shade and 4.9% inside houses (Table V). Proportion clean, slightly dirty, dirty and very dirty nets was 22.4, 35.7, 31.3 and 10.7%, respectively during the terminal survey (Table IV).

**TABLE IV:** Washing frequency and appearance of LLINs

Survey (month)	Number of nets	Mean (SD) number of washes	General aspect of nets (%)			

			Clean	Slightly dirty	Very dirty	Dirty
1	30	0	83.3	13.3	3.3	0
6	30	0.9 (1.1)	40.0	36.7	23.3	0
12	240	1.8 (1.6)	40.9	33.8	21.9	3.4
18	30	1.7 (1.1)	23.3	63.3	10.0	3.3
24	180	4.8 (4.1)	19.4	52.9	18.2	9.4
30	30	4.4 (2.5)	26.7	16.7	50.0	6.7
36	300	8.3 (6.9)	22.4	35.7	31.3	10.7

SD: standard deviation.

**TABLE V:** Drying practice of LLINs

Practice	one week	six months	12 months	18 months	24 months	30 months	36 months

	LLIN %	LLIN % (n = 14)	LLIN % (n = 191)	LLIN% (n = 28)	LLIN% (n = 166)	LLIN% (n = 29)	LLIN% (n = 268)
Drying under sun	0	85.7	52.4	85.7	72.9	51.7	63.8
Drying under shade	0	14.3	34.0	10.7	19.3	48.3	31.3
Inside house	0	0	13.6	3.6	7.8	0.0	4.9

*Physical inspection of nets* - At one month post-distribution, no loss of nets was observed. The proportions of nets that were found lost during the subsequent surveys are given in Table VI. During the survey at 30 and 36 months, the net loss was higher, the maximum (12.6%) was at 36 months post-distribution of nets (Table VI).

**TABLE VI:** Number of LLINs provided/physically present

Nets provided/present	one week	six months	12 months	18 months	24 months	30 months	36 months
No nets provided	30	53	454	57	322	55	564
No of nets found	30	51	435	56	307	50	493
Nets lost	0	2	19	1	15	5	71
Lost (%)	0	3.8	4.2	1.8	4.7	9.1	12.6

During the survey conducted after six, 12, 18, 24, 30 and 36 months of distribution of nets, 6.7% (n = 30), 17.5% (n = 240), 56.7% (n = 30), 25% (n =180), 53.3% (n = 30) and 39% (n = 300) nets were found with holes respectively. A gradual increase was observed in mean hole index from 13.1 ± 49.9 at six months of distribution to 109.1 ± 304.5 at 36 months (Table VII). Similar increasing trend was also observed in mean hole area from 16.1 ± 61.2 at six months of distribution to 133.9 ± 373.8 at 36 months (Table VII). The percentage of nets repaired varied from 0 to 3.3% during the surveys conducted at six to 36 months (Table VIII). Most of the holes present in the nets were found in the lower half (Table VIII).

**TABLE VII:** Physical integrity of LLINs - estimates of the mean hole index and mean hole area

Survey (month)	No. of nets surveyed	Hole index mean (± SD)	Hole area cm^2^ mean (± SD)
6	30	13.1 (49.9)	16.1 (61.2)
12	240	25.3 (128.8)	31.0 (158.1)
18	30	73.6 (137.5)	90.4 (168.8)
24	180	72.0 (251.1)	88.3 (308.3)
30	30	81.3 (144.8)	99.8 (177.8)
36	300	109.1 (304.5)	133.9 (373.8)

± SD: standard deviation.

**TABLE VIII:** Physical integrity of ICON MAXX long-lasting treatment by survey - holes by distribution

Survey (month)	No. of nets surveyed	Distribution of holes on net panels (%)			Mean no. of open seams	Nets with any repairs (%)

		Lower	Upper	Roof		
1	30	0	0	0	0	0
6	30	0	67	33	0.03	0
12	240	58	30	12	0.1	1.3
18	30	58	35	7	0.1	3.3
24	180	53	33	14	0.1	1.7
30	30	34	50	16	0.2	0
36	300	47	43	10	0.1	2

## DISCUSSION

LLIN treatment kit is an improved technology designed to transform untreated nets into LLINs after simple dipping, combining a conventional insecticide with a binding agent (WHO 2007a). Currently, the only available long-lasting insecticide treatment kit is ICON MAXX (WHO 2007a). The safety assessment and review of the efficacy of ICON MAXX in Phase I and Phase II studies were published previously in the report of the 11th meeting of the WHOPES Working Group (WHO 2007c). Taking into consideration the safety, efficacy and resistance to washing of nets treated with ICON MAXX in laboratory and small-scale field studies, it was recommended by WHOPES for large-scale (Phase III) field studies to confirm the long lasting efficacy of the treatment as a requirement for developing full recommendations on the use of the ICON MAXX (WHO 2007c). Subsequent to the above recommendations, the WHOPES facilitated two Phase III trials of polyester nets manually treated at the recommended dosage of lambda-cyhalothrin using the ICON MAXX kit in India and the United Republic of Tanzania to determine the duration of the effective life of the insecticide up to 36 months.

In the current study, after one year of household use, the average lambda-cyhalothrin content in the LLIN and the ITN decreased to 72.9% and 64%, respectively, of their baseline. However, the mortality of *An. fluviatilis*, the major malaria vector in the study area, was 100% on exposure to both the types of nets. Thus, both the LLINs and the ITNs had a comparable insecticide efficacy up to a period of one year of household use. The bio-efficacy of the ICON MAXX treated nets was high through 30 months. At 36 months, bio-efficacy of the nets dropped, and 35 of 80 nets (43.7%) failed according to the cone test. Of the 35 nets that failed the cone test, only two passed the tunnel test for an overall pass rate of 58.8% (47 out of 80). This could be due to the loss of 44.4% of the original concentration of lambda-cyhalothrin content in the LLINs after 36 months of household use. According to WHOPES criteria, at least 80% of the sampled nets have to retain bio-efficacy in cone bioassay or tunnel test at the end of 36 months (WHO 2005, 2013). But, in the current study, only 58.8% of nets met the efficacy criteria and as a result of which the ICON MAXX LLIN was not proved to be effective up to 36 months. In contrary, in Tanzania, where ICON MAXX was tested at Phase III, the results indicated that after 36 months of routine use of nets, 73.7% of the initial lambda-cyhalothrin concentration had been lost. The results of cone bioassay with susceptible *An. gambiae* showed that only 26% of nets met the efficacy criteria after 36 months. But, in the tunnel test more than 80% of the ICON MAXX treated nets met the efficacy criteria at 36 months (WHO 2014). In conclusion, while ICON MAXX treated LLIN retained the bio-efficacy up to 30 months in India, it retained up to 36 months in Tanzania.

The most important finding of the current study was high use rate of the LLINs, with more than 65% of the respondents reporting using the nets year-round and every night, and this could be an indication of people’s willingness for ownership. The use of LLINs was ensured as most LLINs were found hanging inside houses when interviewers visited the houses in morning hours and this could be considered as an evidence for net usage. Those who did not use their nets year-round every night used their nets either occasionally or seasonally. Only 7.3% of respondents reported not using their nets at all during the last 36 months. Thus, ICON MAXX was highly accepted as a defence against nuisance bite of mosquitoes and malaria prevention. In Tanzania trial, the ICON MAXX nets were well accepted by the communities. Reported net use rate was 100% at each time point (WHO 2014). The lesser frequency of net washing in India compared to Tanzania could be the reason for lesser (44.4%) loss of lambda-cyhalothrin content in LLINs in Indian trial after 36 months of household use compared to the higher (73.7%) loss of lambda-cyhalothrin concentration in Tanzania trial.

The estimated rate of washing gradually increased over time during the study from 0.9 per net at the six-month follow-up to 8.3 per net at the 36-month follow-up. In Tanzania trial, it was observed that unlike India, the washing frequency was so high and the mean frequency of washing was estimated to be one to six times per year (WHO 2014). In the current study, one of the reasons for not washing the nets so frequently was that the tribes in the study area received the medicated nets for the first time and fever due to malaria came down drastically during the study period. They were aware of proper usage, adverse effect of frequent washings etc. from the ongoing health education programme of the Community Health Centre. Hence, the villagers did not wash their nets so frequently like Tanzania. All the nets in the current trial were found washed in cold water. Washing nets with commercially available detergent powder was the general practice (98.9%) in the study area. It could be due to the fact that the detergent powders were more commonly available in the local shops/market and less expensive compared to basic soap; thus the villagers preferred to buy detergent powder for net wash. However, washing of treated nets with a detergent powder is not a best practice and WHO recommends the use of an ordinary soap instead. But, the main issues, of course, could be lack of local availability of soap in all villages and proper communication messages in favour of the use of local soap instead of a detergent powder. Drying of nets in shade would be appropriate to preserve the effectiveness of insecticide in the net after washing. This message was given to the households in the villages at the time of distribution of nets to get full benefit of the nets. However, during the survey, it was observed that majority (51.7% to 85.7% in different surveys) of nets were dried in sunlight. This was due to the fact that tribal huts are so small, mostly having single room which is also the kitchen and have no sufficient space inside the hut to tie the wet nets for drying. Therefore, they dried the nets outside the huts in sunlight. This might have enhanced the loss of insecticide content in the LLINs.

The other outcome of the current study was the hole index and hole area of the LLINs increased with the increase of time due to continuous use of nets by the villagers, which is in agreement with the results obtained from a study conducted in Central India (Bhatt et al. 2012). Major causes of wear and tear of the nets were due to rat biting and burns. In spite of all these adversaries, around 61% of the LLINs was found in good condition and 87.4% people kept their nets after three years of use.

Though, the information generated by the current study was useful and relevant for malaria intervention, a few limitations may have influenced it. One limitation would be some recall bias in collection of data on bed net use. But, the respondents were interviewed particularly about bed net use the night before, keeping recall bias to a minimum as found elsewhere (Pettifor et al. 2009). Another limitation would be some recall bias in collection of data on washing practice of bed nets. The respondents were to recollect the number of times they washed their nets since distribution to date of interview which was six months to three years. The other limitation of the study was insufficient number of *An. fluviatilis* used in bioassays conducted at six, 12 and 18 months.

In the current study, the performance of ICON MAXX treated LLINs was examined in Indian field setting. The LLINs were found to retain effective bio-efficacy up to 30 months. However, the desired bio-efficacy could not be sustained up to 36 months. The attrition rate and the percentage of survived nets after 36 months of field use was 12.6% and 87.4%, respectively.
